# Metatranscriptomic Sequencing of Sheath Blight-Associated Isolates of *Rhizoctonia solani* Revealed Multi-Infection by Diverse Groups of RNA Viruses

**DOI:** 10.3390/v16071152

**Published:** 2024-07-17

**Authors:** Michael Louie R. Urzo, Timothy D. Guinto, Ana Eusebio-Cope, Bernard O. Budot, Mary Jeanie T. Yanoria, Gilda B. Jonson, Masao Arakawa, Hideki Kondo, Nobuhiro Suzuki

**Affiliations:** 1 Microbiology Division, Institute of Biological Sciences, College of Arts and Sciences, University of the Philippines Los Baños, Los Baños 4031, Laguna, Philippines; mrurzo@up.edu.ph (M.L.R.U.); tdguinto@up.edu.ph (T.D.G.); 2 Fit-for-Future Genetic Resources Unit, Rice Breeding Innovations Department, International Rice Research Institute (IRRI), University of the Philippines Los Baños, Los Baños 4031, Laguna, Philippines; 3 Institute of Weed Science, Entomology, and Plant Pathology, College of Agriculture and Food Science, University of the Philippines Los Baños, Los Baños 4031, Laguna, Philippines; bobudot@up.edu.ph; 4 Traits for Challenged Environments Unit, Rice Breeding Innovations Department, International Rice Research Institute (IRRI), University of the Philippines Los Baños, Los Baños 4031, Laguna, Philippines; m.yanoria@irri.org (M.J.T.Y.); g.jonson@irri.org (G.B.J.); 5 Faculty of Agriculture, Meijo University, Nagoya 468-8502, Japan; aramasa@meijo-u.ac.jp; 6 Plant-Microbe Interactions Group, Institute of Plant Science and Resources (IPSR), Okayama University, Chuo 2-20-1, Kurashiki 710-0046, Japan

**Keywords:** *Rhizoctonia solani*, dsRNA, mycovirus, RNA virus, metatranscriptome

## Abstract

Rice sheath blight, caused by the soil-borne fungus *Rhizoctonia solani* (teleomorph: *Thanatephorus cucumeris,* Basidiomycota), is one of the most devastating phytopathogenic fungal diseases and causes yield loss. Here, we report on a very high prevalence (100%) of potential virus-associated double-stranded RNA (dsRNA) elements for a collection of 39 fungal strains of *R. solani* from the rice sheath blight samples from at least four major rice-growing areas in the Philippines and a reference isolate from the International Rice Research Institute, showing different colony phenotypes. Their dsRNA profiles suggested the presence of multiple viral infections among these Philippine *R. solani* populations. Using next-generation sequencing, the viral sequences of the three representative *R. solani* strains (Ilo-Rs-6, Tar-Rs-3, and Tar-Rs-5) from different rice-growing areas revealed the presence of at least 36 viruses or virus-like agents, with the Tar-Rs-3 strain harboring the largest number of viruses (at least 20 in total). These mycoviruses or their candidates are believed to have single-stranded RNA or dsRNA genomes and they belong to or are associated with the orders *Martellivirales*, *Hepelivirales*, *Durnavirales*, *Cryppavirales*, *Ourlivirales*, and *Ghabrivirales* based on their coding-complete RNA-dependent RNA polymerase sequences. The complete genome sequences of two novel RNA viruses belonging to the proposed family *Phlegiviridae* and family *Mitoviridae* were determined.

## 1. Introduction

The Philippines occupies the sixth place among the rice-consuming countries in the world and is one of the countries where per capita rice consumption is increasing [1]. On a global scale, one formidable opponent in achieving maximum rice yield potential is losses due to pests and diseases. These losses can reach up to 37%, of which 11–13% are attributed to fungal pathogens [2]. Rice sheath blight caused by the soil-borne fungus cosmopolitan *Rhizoctonia solani* (teleomorph: *Thanatephorus cucumeris*) contributes immensely to this yield loss. In the Philippines alone, it accounts for a yield loss of 0–48% and 20–42% depending on rice varieties and varying nitrogen fertilizer rates applied [3,4]. This fungus reduces the productivity of tillers by producing visible lesions with brown margins and a grayish center on infected leaves running through the sheaths of rice plants. Sclerotia (dormant structures or aggregated hypha) emerging from mycelial networks are formed and distributed in the infected plant parts where they persist in soil and later become a source of infection for the next disease cycle [5]. The success of this soil-waterborne fungus may be attributed to its properties of being highly variable in terms of morphology, host range, virulence, and genetics [6,7]. Together with its soil-borne nature, the lack of resistant rice varieties makes this fungus difficult to control. Taxonomically, *R. solani* is classified under the phylum Basidiomycota [7]. Variability with respect to hyphal interaction categorizes *R. solani* into 14 anastomosis groups (AG; AG1 to AG13 and AG-BI) each having related but unique sub-groups or intraspecific groups (ISGs) [8,9,10,11,12].

Mycoviruses (fungal viruses) infect fungal species or fungus-like organisms, and have been reported in all the major phyla of fungi, including the Chytridiomycota, Zygomycota, Ascomycota, Deuteromycota, and Basidiomycota [13,14]. Importantly, mycovirus studies have contributed to the expansion of the virosphere as well as the discovery of new virus lifestyles [13]. Generally, most mycoviruses cause asymptomatic infections, while some induce observable phenotypic alterations including hypovirulence when they infect pathogenic fungi [15,16,17]. Over 100 viruses have been reported from *R. solani*, including positive-sense (+) single-stranded RNA (ssRNA), negative-sense (–) ssRNA, and double-stranded RNA (dsRNA) viruses [18,19]. Of note is that a plant virus, cucumber mosaic virus (CMV), was found to naturally infect *R. solani* through cross-kingdom viral transmission [20]. *Rhizoctonia* spp. often show multiple virus infections and high prevalence rates [18,21,22,23]. While most of them are assumed to be asymptomatic like most other fungal viruses, some induce phenotypic alterations. For example, Rhizoctonia solani endornavirus 1 (RsEV1) [24] and Rhizoctonia solani partitivirus 2 (RsPV2) [25] have been shown to reduce the virulence of the host fungus *R. solani.* Furthermore, *Rhizoctonia* mycoviruses have been implicated in changing their host fungal susceptibility to fungicides [26], as in other fungal host/mycovirus systems (e.g., [27]).

While mycoviruses hosted by both non- and phytopathogenic *Rhizoctonia* spp. have been extensively studied throughout the world, no such study has been conducted for the Philippine collections. As a first step towards a comprehensive understanding of mycoviruses in the Philippine *Rhizoctonia* populations, we targeted a collection of *R. solani* strains collected from different islands for dsRNA screening and conducted the virome analysis of three selected *R. solani* strains among them. We identified multiple novel mycoviruses infecting field strains based on metatranscriptomic sequencing and further validated them by RT-PCR. Our findings provide evidence for the multiple infections of a single *R. solani* isolate by up to 20 or more mycoviruses spanning 12 different viral families.

## 2. Materials and Methods

### 2.1. Collection of Field-Infected Rice Material for Isolation of Rhizoctonia solani

**Figure 1:**
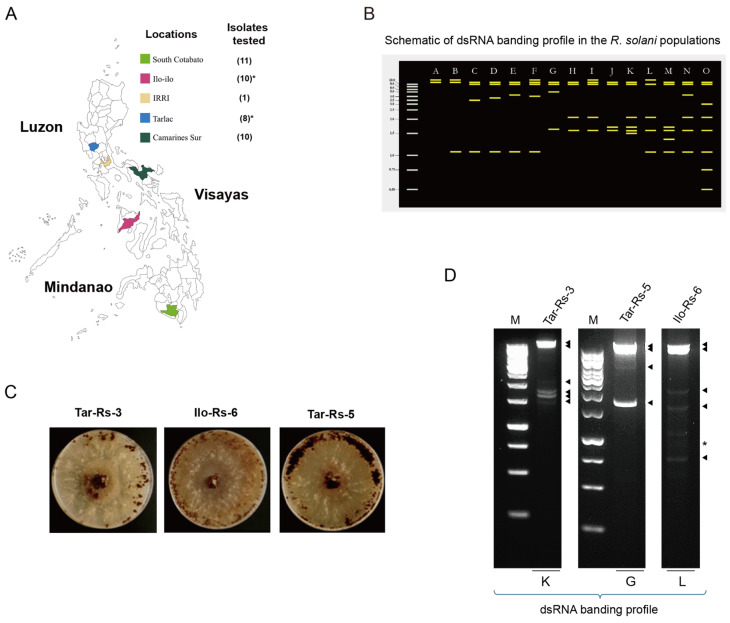
*R. solani* strains used for the dsRNA screening and subsequent virome analysis. (**A**) The collection sites of *R. solani* strains in different parts of the Philippines. The map shows the regions where sheath blight-infected rice samples were collected, covering the rice-producing regions of Luzon, Visayas, and Mindanao, the three main islands of the country. The number of tested *R. solani* strains collected from each sampling region for this study is shown in parentheses. Asterisks include the fungal strains used for the metatranscriptomic sequencing and further characterization of the mycoviruses. (**B**) The schematic categorization of the dsRNA banding profiles. The dsRNA gel electrophoretic patterns shown in Figure S3 were utilized. Potentially, the fifteen banding profiles (labeled A to O) may contain from two high molecular weight dsRNA bands (A) to eight bands of different sizes (O). (**C**) The fungal colony morphology of the three selected *R. solani* strains (Tar-Rs 3, Ilo-Rs 6, and Tar-Rs 5). The three strains were grown on oatmeal agar for 10 days in 90 mm plates under continuous darkness at 28 °C. (**D**) The dsRNA banding profile of the three fungal strains. The dsRNA fraction obtained from each strain was electrophoresed in 1.4% agarose gel at 100 V for 45 min and stained with ethidium bromide. * refers to rRNA. The labels J, G, and L show the categorization based on the respective dsRNA profiles (see Figure S3).

Rice plants showing the typical symptoms of sheath blight were collected from at least 4 rice-growing areas in the Philippines. The collection sites were representative of the three biggest islands in the country: Luzon, Visayas, and Mindanao (see Figure 1A and Figure S1). The specimens were brought to the Institute of Weed Science, Entomology, and Plant Pathology (IWEP), the College of Agriculture and Food Science (CAFS), University of the Philippines Los Baños (UPLB) for the isolation of *Rhizoctonia* spp. strains (Figure S1). A total of 39 *Rhizoctonia* spp. strains were obtained from infected field materials. Additional *Rhizoctonia* sp. strain from the Plant Pathology and Host Plant Resistance Group Laboratory, Rice Breeding Innovations (RBI) Department, the International Rice Research Institute (IRRI) was included as a reference (see Figure S1 for an isolation method for fungal strains). These fungal strains were identified as *R. solani* based on their isolation host plant, rice, symptomatology in rice plants, and thorough morphological examination (Table S1 and Figure S2). A total of 40 *R. solani* strains were evaluated (Table S1).

Among these *R. solani* strains, the three of interest, Tar-Rs-3, Ilo-Rs-6, and Tar-Rs-5, were further validated through a ribosomal internal transcribed spacer (ITS) region sequencing. Using ITS1 and ITS4 primers, two ITSs and their intervening 5.8S ribosomal RNA gene were amplified [28] and subsequently cloned into the pGEM^®^T-Easy Ligation Vector (Promega, Madison, WI, USA). Direct Sanger sequencing was performed on amplicons from colony PCR with positive bacterial colonies.

The cultures of the tested strains of *R. solani* were stored on potato dextrose agar (PDA, BD Difco, Franklin Lakes, NJ, USA) slant (150 mm × 16 mm with 14 mm inner diameter) and in Petri plates (90 mm diameter) at 4 °C until use.

### 2.2. Phenotype of Rhizoctonia spp. on Artificial Culture Media

Ten-day-old fungal cultures of *Rhizoctonia* spp. were assessed for phenotypic differences focusing on colony color, the growth pattern based on mycelium production, and sclerotial variability [29]. The *Rhizoctonia* spp. were grown simultaneously in different growth media: PDA and oatmeal agar for cultural characterization. The strains were incubated under the same laboratory conditions, specifically at 28 °C in continuous darkness, until their phenotypes were observed. The observations were recorded, and the fungal strains were documented and photographed.

### 2.3. dsRNA Screening among Rhizoctonia solani Collections

Subculturing on PDA plates overlaid with cellophane membranes (cat. #. 89196457; pore size PT#300; 30 g/m^2^, Heiko Pack, Ltd., Haga, Tochigi, Japan) was used to propagate the mycelia of fungal strains for dsRNA screening. Four-day-old mycelial growth of *Rhizoctonia* spp. was harvested from the cellophane covering the top portion of each PDA plate. About 3–5 pieces of cellophane (~1 g mycelial) per isolate were collected and evaluated for dsRNA content following the dsRNA extraction procedure of Eusebio-Cope and Suzuki [30]. Briefly, mycelia were pulverized using mortar and pestle with liquid nitrogen. The powder was covered with a sufficient volume of phenol:chloroform:isoamyl-alcohol (25:24:1, *v*/*v*), mixed in the container, and was transferred to a 2 mL microcentrifuge tube. Following centrifugation at 14,000 rpm, the supernatant was further clarified with chloroform:isoamyl alcohol, and the dsRNA was selectively purified using cellulose powder (cellulose powder B3; Advantec Co., Ltd., Tokyo, Japan). The obtained fractions were then double digested by RQ1 RNase-free DNase I (Promega) and S1 Nuclease (Takara Bio Inc., Kusatsu, Shiga, Japan). Elution and precipitation were conducted using 3 M sodium acetate (pH 5.2). The presence of dsRNA bands in the agarose gel electrophoresis was recorded as an indication of possible virus infection.

Attempts to cure the fungal strains involved the use of the combined techniques of an antiviral chemical (ribavirin; product code R9644-10MG, Sigma-Aldrich, Co., St. Louis, MO, USA) and a hyphal tip-cutting technique. One hundred microliters of 0.08 M ribavirin was added to a PDA plate on which the strains of *R. solani* strains of interest (Tar-Rs-3, Ilo-Rs-6, and Tar-Rs-5) were grown for 3 days before the hyphal tip was transferred to a new plate. The method was performed in three successive trials.

### 2.4. Viral RNA Sequencing and Bioinformatic Analyses

Virus-associated sequences were obtained by the next-generation sequencing (NGS) of total RNA fractions extracted from the mycelia of three selected *R. solani* strains (Tar-Rs-5, Ilo-Rs-6, and Tar-RS-3). Total nucleic acid was extracted with phenol-chloroform and two rounds of chloroform purification, followed by sodium acetate precipitation. Host DNA was digested with DNase I (Promega) following the manufacturer’s procedures. A pooled sample of the total RNA fraction from the Tar-Rs-5, Ilo-Rs-6, and Tar-Rs-3 strains was sent to Macrogen Japan Co. (Tokyo, Japan) for 100 base pair (bp) paired-end sequencing using an Illumina NovaSeq 6000 after ribosomal RNA depletion (Ribo-Zero kit, Illumina, San Diego, CA, USA) and library preparation (TruSeq Stranded Total RNA LT Sample Prep Kit-Human, Mouse, Rat) [31]. Raw sequencing reads (42.3 M) were processed using the CLC Genomics Workbench (version 11, CLC Bio-Qiagen, Aarhus, Denmark). Briefly, adaptor sequences were trimmed and contig sequences were assembled de novo, resulting in 24,565 contigs. A local BLASTx [32] search was run to compare the contigs to viral reference sequences (RefSeq) from the National Center for Biotechnology Information (NCBI) [33]. As an alternative analysis, the SPAdes assembly program [34] was also used with 10.9 million clean reads after the removal of host-associated reads to generate the contig assembly. This was followed by a BLASTx analysis using a viral sequence dataset downloaded from Viral NCBI (accessed on 1 April 2023) [32,33,34]. To estimate the abundance of each virus, virus-derived RNA-seq reads were normalized using fragments per kilobase of transcript per million fragments mapped (FPKM) in the CLC Genomics Workbench. The identified viral sequences obtained from the SPAdes assembly were deposited in GenBank/EMBL/DDBJ with the accession numbers listed in Table S2 (and see also Table 1).

Protein domain searches were performed in the NCBI Batch Web CD-Search Tool (https://www.ncbi.nlm.nih.gov/Structure/bwrpsb/bwrpsb.cgi; accessed on 31 March 2024) and visualized with the package gggenes using a custom script in R (https://wilkox.org/gggenes/ (accessed on 14 January 2024)) [35,36].

The phylogenetic characterization of the sequences was based on sequence alignment of the RNA-dependent RNA polymerase (RdRP) region of each virus detected and confirmed in the fungal isolates. Representative sequences were obtained from NCBI and alignment was performed using the MAFFT online server version 7 (https://mafft.cbrc.jp/alignment/server/index.html; accessed on 14 January 2024) [37]. A maximum likelihood tree was constructed using IQ-TREE web server (http://iqtree.cibiv.univie.ac.at/; accessed on 14 January 2024) with 10,000 replicates of ultrafast bootstrap analysis and the SH-aLRT branch test and automatic substitution model selection based on Akaike information criterion (AIC) scores predicted by ModelFinder [38,39] unless otherwise mentioned. All the trees were viewed and annotated using the online platform iTOL v6 online tool (https://itol.embl.de/; accessed on 14 January 2024) [40].

A Sankey diagram was generated using SankeyMATIC by Steve Bogart (https://sankeymatic.com/; accessed on 19 January 2024), connecting fungal strains to the respective taxonomic classification of the identified virus candidates, starting with the viral families and up to the realm to which they belong.

The terminal sequences of RsPhV1 and RsMV41 were determined by the 5′ RNA ligase-mediated rapid amplification of cDNA ends (RLM-RACE) and Sanger sequencing according to a previously established method [31]. Briefly, the dsRNA of Tar-Rs-3 and Ilo-Rs-6 were extracted as described above and used for RLM-RACE. cDNA covering the terminal ends were amplified using the adapter and specific primers listed in Table S4. The consensus sequence of each terminus was obtained based on the alignment of at least five independently sequenced clones. The alignment of the regions was performed using the GENETYX^®^-MAC Network Ver. 21 software (GENETYX Corp., Tokyo, Japan).

### 2.5. RT-PCR Validation of Viruses in Fungal Strains

The infection of each viral candidate was validated by the amplification of the specific regions of viral genomic RNA. Briefly, the total RNA of the *R. solani* strains Tar-Rs-5, Ilo-Rs-6, and Tar-RS-3 was extracted as described above, and each virus or viral segment was detected individually. Using a 1:10 dilution of the total RNA extract, one-Step RT-PCR was performed using the Takara One-Step RT-PCR Kit (Takara Bio) according to the manufacturer’s protocol. Amplicons were run on 1% agarose gel electrophoresis at a constant 100 V for 30 min, followed by staining with ethidium bromide. Only those with proper amplification by RT-PCR were considered to be a confirmed virus infection. Primers used for virus detection are summarized in Table S3.

## 3. Results

### 3.1. The Common Colony Phenotypes and Unique dsRNA Profiles in the Rhizoctonia *spp.* Collection

Among the 40 Philippine *Rhizoctonia* spp. strains subjected to this study, we obtained 8 isolates from Tarlac (Tar-Rs-1 to Tar-Rs-8), 10 isolates from Camarines Sur (Cs-Rs-1 to Cs-Rs-10), 10 isolates from Iloilo (Ilo-Rs-1 to Ilo-Rs-10), 11 isolates from South Cotabato (Sc-Rs-1 to Sc-Rs-11), and 1 strain from the IRRI (Figure 1A and Table S1). When grown on PDA and oatmeal agar, they showed significant differences in sclerotial body formation (Figure S2). Compacted ring patterns adjacent to the inoculation site were more common for the strains when grown on oatmeal agar, while we observed a loosely scattered formation or highly compacted sclerotial bodies at the inoculation site on the PDA plates. The color was brown to dark brown, consistent with the previous studies of *R. solani* [6] (Figure S2). In this study, the 40 Philippine fungal isolates were hereafter referred to as *R. solani* based on their original symptoms in the host plants (rice) and their colony morphology on the media.

Of the 40 *R. solani* strains tested for dsRNA screening, surprisingly, we detected potential virus-associated dsRNA elements in all the dsRNA-enriched samples of these strains (Figure S3). The dsRNA banding profiles showed at least 15 unique combinations of different dsRNA sizes (Figure 1B and Figure S3, categories A to O). We detected high-molecular-mass dsRNA bands—approximately 8 kilobases (kb) up to >10 kb—in all the samples tested; we also found lower-molecular-weight dsRNA bands (e.g., ~1.1 kb) in several strains (see Figure S3). The patterns A and L are predominant in isolates from Camarines Sur and Iloilo, respectively, while isolates from Tarlac and South Cotabato have varying dsRNA banding profiles. Despite using almost the same amount of fungal tissue samples as starting materials, the visible dsRNA bands showed different intensities, which can be attributed to the different viral titers in each fungal strain. This is prominent in the *R. solani* strains Ilo-Rs-3 and Ilo-Rs-4, which had very weak high-molecular-weight bands compared with the other strains collected from the same rice production area (Figure S3). Among these fungal collections, we subjected three representative *R. solani* strains (Tar-Rs-3, Ilo-Rs-6, and Tar-Rs-5) with dsRNA banding patterns categorized as K, L, and G, respectively, to mycovirome analysis (Figure 1C,D) because they contain bands of similar size that appear to be categorized in other strains, including bands that are unique to a specific strain (Figure 1B and Figure S3).

### 3.2. Virus Population in the Three Philippine R. solani Strains

**Figure 2:**
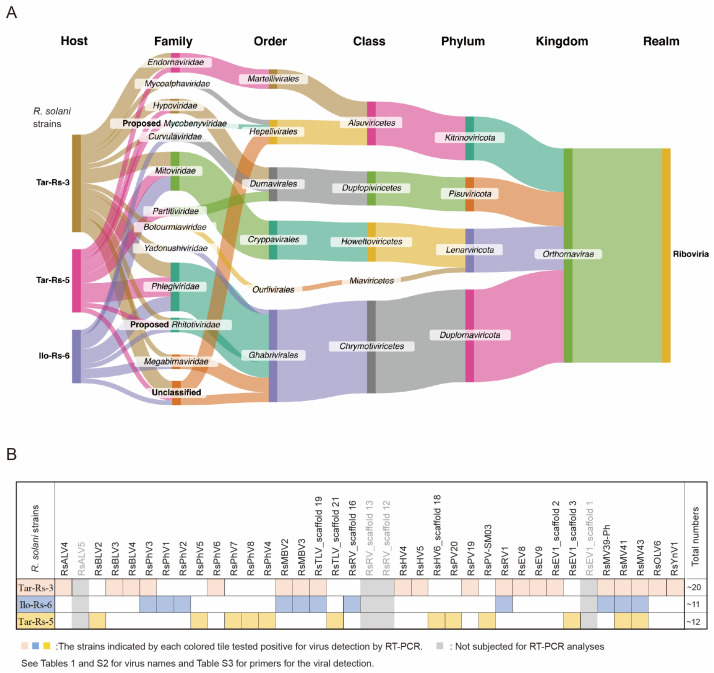
Summary of mycoviruses infecting selected *R. solani* strains. (**A**) The Sankey diagram generated using Sankeymatic. The flow diagram highlights the key general taxonomic classification of all the viruses found in the metatranscriptomic data from the three utilized *R. solani* strains (Tar-Rs 3, Ilo-Rs 6, and Tar-Rs 5). The fungal strains are connected to the respective taxonomic classification of the identified viruses starting with the viral families up to the realm in which they belong. The columns represent the taxon while the flow width indicates the relative number of viruses in each grouping. (**B**) The virus detection patterns in the three *R. solani* strains. The tile diagram shows which virus was found in a particular strain, with the colored tiles corresponding to a positive result as summarized in Figure S3. RT-PCR targeting RdRP or a specific coding region within the viral genome was used for the verification and host identification of the viral sequences (Table S3 for the list of primer sets for each virus).

After the RNA sequencing of a pooled total RNA sample and the data processing of the obtained raw reads, at least 42 virus-related contig sequences are summarized in Table 1 and Table S2. Many of them are presented with a complete coding sequence (CDS), and some contig sequences are variants of the same virus species. We grouped the viruses and identified their respective taxonomic classification (Figure 2A) according to 15 hierarchical ranks of virus classification set by the International Committee on Taxonomy of Viruses Executive Committee (ICTV). All the viral sequences were from a single kingdom, *Orthornavirae* of the realm *Riboviria*, which have RNA genomes. Grouping all the viruses together, four phyla emerged as likely to infect the *R. solani* strains, with *Duplornaviricota* having the highest number of viruses as represented by viruses from the order *Ghabrivirales* indicated by the height of the bars (Figure 2A). Because the submitted sample was a pool of total RNA from three *R. solani* strains, we determined the accompanying virus (some contigs were not subjected to analysis) in each host using RT-PCR. There are at least 20, 11, and 12 different viruses potentially infecting the Tar-Rs-3, Ilo-Rs-6, and Tar-Rs-5 strains, respectively (Figure 2B and Table S2). The flow diagram also suggests that the host with the most diverse infecting viruses is the Tar-Rs-3 strain, which harbors five unrelated viral classes, while the Ilo-Rs-6 strain shows the least diversity with only three different classes in it. Meanwhile, there are several virus candidates from the three fungal strains that may belong to unclassified viral families (Figure 2A).

Attempts to cure the selected strains of the infecting mycovirus (the Tar-Rs-3, Ilo-Rs-6, and Tar-Rs-5 strains) with the antiviral drug ribavirin failed to produce dsRNA-free daughter strains. To date, only *Rhizoctonia oryzae-sativae* of the genus *Rhizoctonia* has been successfully cured of an infecting mycovirus [41], and thus our tested fungal strains may have a much higher viral fitness than that of the previous study.

### 3.3. Viruses of the Order Hepelivirales, Phylum Kitrinoviricota

**Figure 3:**
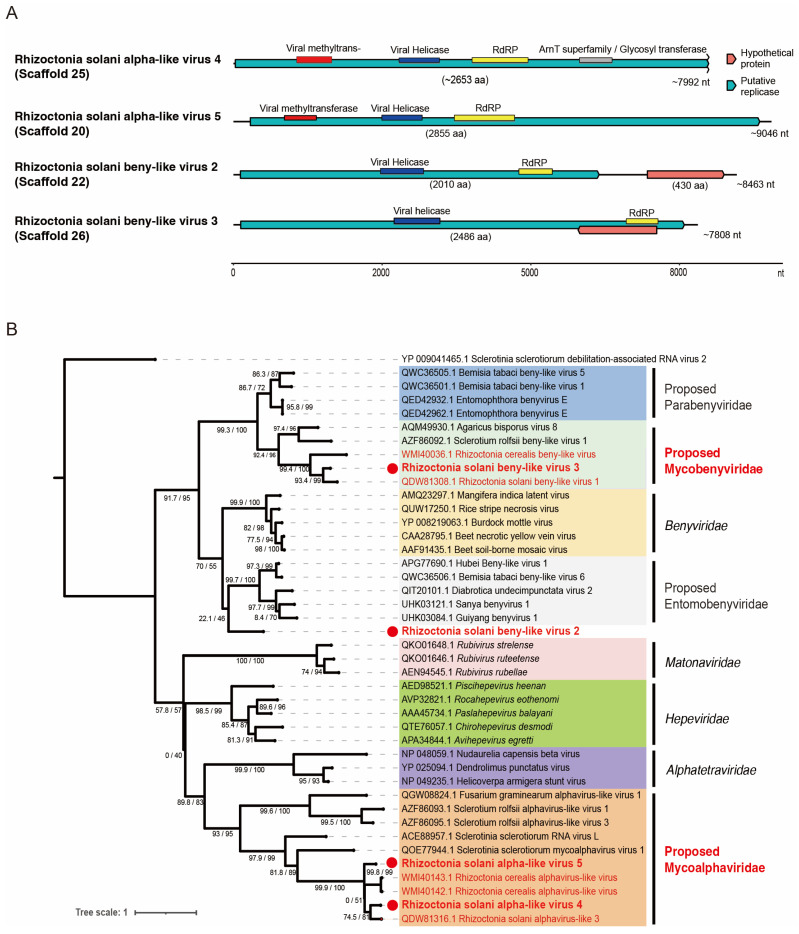
Genome organization and phylogenetic relationships of viruses belonging to the order *Hepelivirales.* (**A**) The genome architecture of the viruses discovered in this study. The black line indicates the determined viral genomic sequence, while the boxes filled with different colors (blue or orange) denote the coding regions in this and the subsequent figures. Functional domains such as RdRP and RNA helicase are shown with different colored bar-boxes. A size scale bar is present at the bottom in this and the subsequent figures. (**B**) The maximum likelihood tree based on the RdRP sequences. The RdRP sequence alignment was performed as described in the Materials and Methods. The resulting alignment was used to generate the maximum likelihood tree using the IQ-TREE webserver with 10,000 replicates of ultrafast bootstrap analysis and SH-aLRT branch test [38]. The best-fit substation model was rtREV+F+I+G4 based on the results of ModelFinder according to the Bayesian information criterion. Clades within the tree are colored based on the viral families. In this and the subsequent trees, the *Rhizoctonia* viruses newly discovered in this study are shown in bold red letter after a red circle. Sclerotinia sclerotium debilitation-associated RNA virus 2 (a tymo-like mycovirus, order *Tymovirales*) was used as the outgroup.

The refined assembly of the metatranscriptomic data revealed five virus-like sequences related to the alpha-like viruses or beny-like viruses of the order *Hepelivirales* ([+]ssRNA viruses), which we tentatively named Rhizoctonia solani alpha-like virus 4 and 5 (RsALV4 and RsALV5; scaffolds 25 and 20) and Rhizoctonia solani beny-like virus 2, 3, and 4 (RsBLV2, RsBLV3, and RsBLV4; scaffolds 22, 26, and 269), respectively (Table 1 and Table S2). A complete CDS of the genome was probably obtained for RsALV5, while the RsALV4 lacks the sequences at the 3′-terminal region, including the stop codon (Figure 3A). On the other hand, both RsBLV2 and RsBLV3 have the complete CDS of their polymerases, while RsBLV2 also encodes an additional open reading frame (ORF) for a hypothetical protein (Figure 3A). RsBLV4 is only 1026 nucleotides long and may be a small portion of a truncated polymerase protein.

We analyzed RsALV4 and RsALV5 by constructing the phylogenetic trees of exemplar and related viruses within the order *Hepelivirales* using their RdRP sequences. Both RsALV4 and RsALV5 cluster within the previously proposed family “*Mycoalphaviridae”* and form a subclade with ~47% amino acid sequence similarity to their closest relatives from *Rhizoctonia* spp. (Table 1) [18]. Despite being closely related based on phylogenetic analysis and sequence homology, taxonomic placement at the genus and species levels is not yet clear, as the demarcation of species within the family has not yet been established and it is not yet a recognized family within the order *Hepelivirales* [42].

A comparison of the RsBLV2 and RsBLV3 RdRP proteins showed that they are only 25% similar to each other. Comparing these beny-like viruses with their closest relatives, RsBLV2 is only 35% similar to Bemicia tabaci beny-like virus 6 and RsBLV3 is 47% identical to Rhizoctonia solani beny-like virus 1 (RsBLV1) (Table 1). With the RdRP sequence similarity and phylogenetic clustering, RsBLV2 and 3 clearly belong to a single large clade, including an established plant virus family *Benyviridae* and other proposed related family candidates; the node has a good support value (Figure 3B). RsBLV3 is placed in the proposed family “*Mycobenyviridae*” together with beny-like mycoviruses, while RsBLV2 is clearly separated from other beny-like viruses as an outlier, forming its own clade that has yet to be identified—more sequences of related viruses may be discovered in the future—so it remains unclassified. The species of the family *Benyviridae* are distinguished by coat protein (CP) sequences that share 90% identity [43], but it is not known whether RsBLV2 and RsBLV3 and other related beny-like mycoviruses have the CP gene. The species demarcation criteria for the proposed families accommodating beny-like viruses need to be set.

### 3.4. Viruses of the Order Martellivirales, Phylum Kitrinoviricota

**Figure 4:**
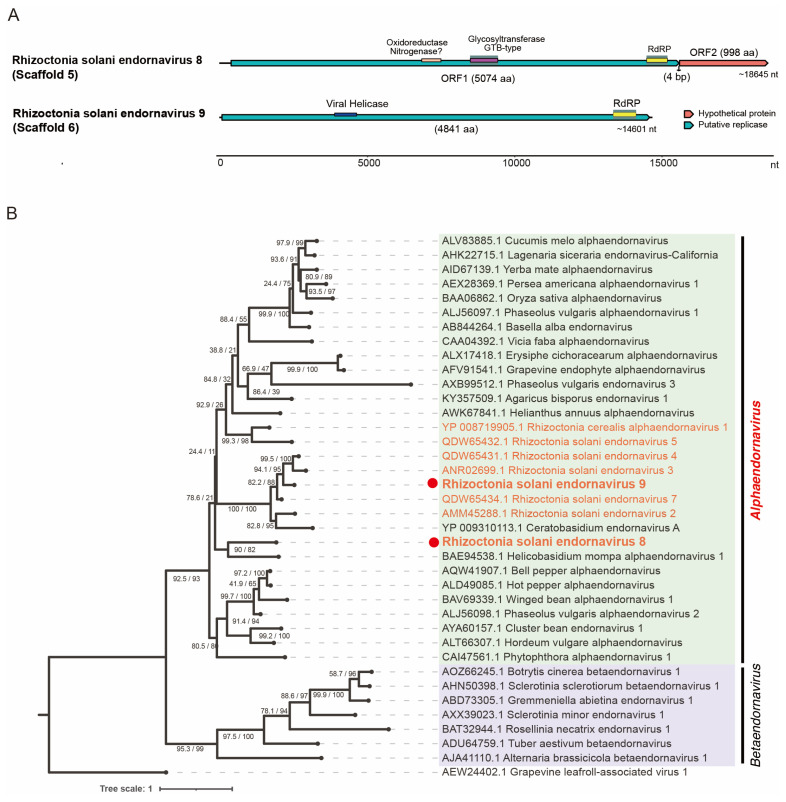
Genomic and phylogenetic analyses of the newly detected viruses belonging to the family *Endornaviridae*, order *Martelivirales*. (**A**) The schematic diagram of the genomes of two endornavirids discovered in this study. (**B**) The maximum likelihood tree generated based on the RdRP sequences. Different genera are shown in different colors. The phylogenetic analysis was run for 10,000 replicates using the ultrafast mode calculating the bootstrap percentage and SH-aLRT with the LG+I+G4 substitution model according to the Bayesian information criterion calculated by ModelFinder. Grapevine leafroll-associated virus 1 (a plant closterovirid, order *Martellivirales*) was used as the outgroup.

Five of the large assemblies (14.6~20.1 kb) are related to the (+)ssRNA genome of the members within the family *Endornaviridae* (order *Martellivirales*). Three endornavirus-like sequences (scaffolds 1, 2, and 3) are related to each other (~91% amino acid sequence identity) and also with Rhizoctonia solani endornavirus 1 (RsEV1, an unclassified endornavirus; ~82% amino acid sequence identity), and thus we tentatively consider these endornavirus-like sequences to be variants of RsEV1 [24] (Table 1). Two other endornavirus-like sequences (scaffolds 5 and 6) are potentially associated with novel endornaviruses, and thus we tentatively named them Rhizoctonia solani endornavirus 8 and 9 (RsEV8 and RsEV9), respectively (Table 1). The genome architecture of RsEV8 shows two non-overlapping ORFs located in two different reading frames with a 4 bp intergenic spacer (Figure 4A). The ORFA protein (5074 amino acids) of RsEV8 contains the RdRP and glycosyltransferase domains, while ORFB (998 amino acids) likely encodes a hypothetical protein with an unknown function. In contrast, RsEV9 has a typical endornavirus-like genome structure with a single large ORF that can encode a protein (4841 amino acids) with the RdRP and viral helicase domains (Figure 4A) [44]. In the BLAST analysis, RsEV8 and RsEV9 were related to RsEV1 and Rhizoctonia solani endornavirus 7 (45% and 39% aa sequence identity, respectively).

To determine the phylogenetic relationship of RsEV8 and RsEV9, we constructed a phylogenetic tree of the family *Endornaviridae* including the sequences of the exemplar isolates (Figure 4B). Both RsEV8 and RsEV9 cluster with alphaendornaviruses, which is also consistent with the current genus demarcation criteria for the family, including the proposed genome size of >11.9 kb for alphaendornaviruses [44]. In addition, the sequence similarity analysis shows that both RsEV8 and RsEV9 likely meet the species demarcation criteria (>75% nucleotide sequence similarity) for novel species in the genus *Alphaendornavirus* [44]. The current description of the family *Endornaviridae* states that its members have only a single large protein-coding gene, but this is not true for RsEV8 and some other endornaviruses such as RsEV1 and *Ceratobasidum* endornaviruses. Hence, there should be a future revision of the description to reflect the diversity in the genome structure of the members of the family *Endornaviridae* [24,45].

### 3.5. Viruses of the Order Ghabrivirales, Phylum Duplornaviricota

We based our taxonomic considerations of the currently known members of the order *Ghabrivirales* on the proposed revision to this order [46]. We discovered a total of eight new viruses related to phlegivirids and tentatively designated them as *Rhizoctonia solani* phlegivirus virus 1 to 8 (RsPhV1–8), corresponding to scaffolds, 9, 10, 15, 14, 7, 8, 24, and 40, respectively (Table 1 and Table S2). Two scaffolds (31 and 27) are related to megabirnavirids and are tentatively named Rhizoctonia solani megabirnavirus 2 and 3 (RsMBV2 and 3), respectively (see detail below). We also identified three scaffolds (16, 13, and 12) that are significantly similar to an unpublished megabirna-like virus (Rhizoctonia solani RNA virus, RsRV; 7596 bp, appears to lack ~3kbp in the 5′ region) [47] and are likely to belong to the same species (Table 1). We tentatively assigned scaffolds (19 and 21) as Rhizoctonia solani toti-like virus 1 (RsTLV1), which are closely related to a recently deposited RsTLV1 isolate from China (OR762576). Lastly, we nominally assigned scaffold 377 as Rhizoctonia solani yadonushi virus 1 (RsYn1). We did not include scaffolds 40 (RsPhV8), 377 (RsYnV1), and 27 (RsMBV3) in the subsequent analyses due to the lack of an RdRP sequence, although they were detected in hosts by RT-PCR, as well as the known megabirna-like virus, RsRV (Figure 2B and Table S2).

**Figure 5:**
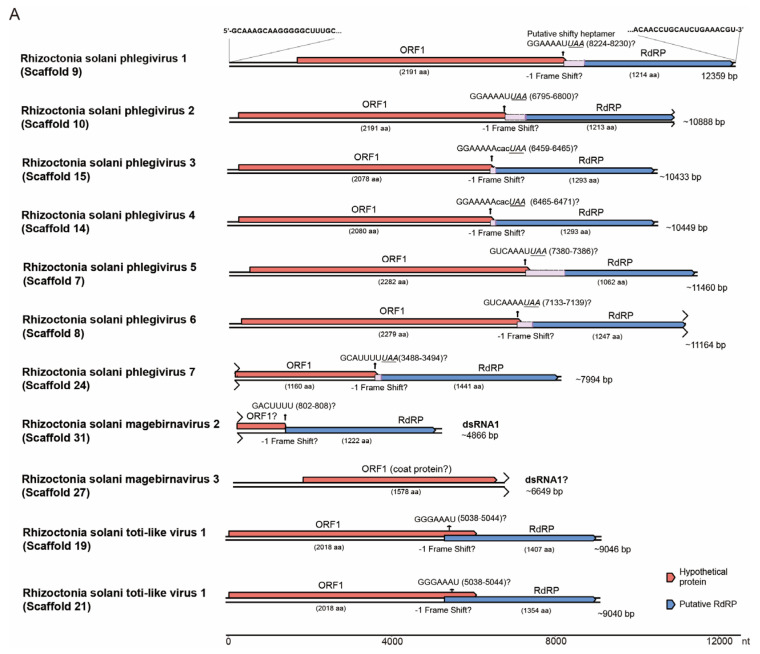

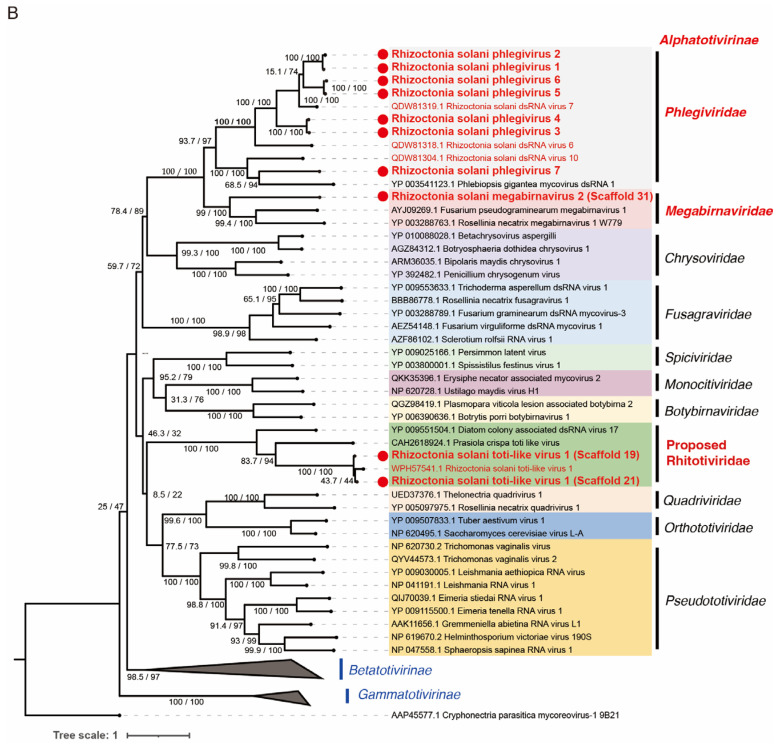
Genomic and phylogenetic analyses of newly detected viruses belonging to the order *Ghabrivirales.* (**A**) The genome organization of the viruses discovered in this study. At least 10 dsRNA viruses—7 phlegivirids (4 novel viruses with their three variants), 1 megabirnavirid, and 2 rhitotivirids (two putative variants)—were detected. The shifty heptamer sequence, which is assumed to be located upstream of the ORF1 stop codon (italicized and underlined; for phlegivirids only), and its relative nucleotide position are shown on each genome (scaffold). (**B**) The maximum likelihood tree generated based on the RdRP sequences. Different family members are shown by different colors. Note that the order *Ghabrivirales* has recently been reorganized by the ICTV [46]. The LG+F+I+G4 substitution model was selected by ModelFinder based on the Bayesian information criterion. Mycoreovirus 1 (a dsRNA mycovirus, order *Reovirales*) was used as the outgroup.

RsPhV1 to RsPhV7 appear to have complete CDSs for their RdRPs with the exception of RsPhV2 and 6 that are truncated at the 3′-terminal regions, and are all closely related to phlegivirids, specifically a Rhizoctonia solani dsRNA virus (Rhizoctonia solani dsRNA 7) and Phlebiopsis gigantea large virus 1 (PgLV1) (Figure 5A and Table 1) [46,48,49]. We have provided the complete sequence of RsPhV1 with its termini through an RLM-RACE analysis (Figure 5A). The genomes of RsPhV1 to RsPhV7 likely contain two ORFs, which encode a hypothetical protein and RdRP in different reading frames. Some studies have suggested the fusion expression of ORF1 and ORF2 for phlegiviruses via –1 ribosomal frameshifting involving a shifty heptamer sequence (X XXY YYZ, where X is A, U, or G; Y is A or U; and Z is A, U, or C) [48,50]. RsPhV1 to RsPhV7 appeared to lack a typical shifty heptamer sequence, while alternative candidates were speculated to potentially cause frameshifting upstream of the stop codon of the ORF1 and downstream of the in-frame stop codon before the AUG of ORF2 (their intervals ranging from 7 to 64 nucleotides) (see Figure 5A).

Scaffolds 31 and 27 likely encode a megabirnavirid RdRP and a megabirnavirid coat protein, respectively (Table 1). The separation of the RdRP and CP coding domains into two genomic segments is atypical for megabirnavirids, wherein these proteins are typically encoded in dsRNA1 and expressed via a slippery sequence/shifty heptamer like totiviruses [51] and those discussed above for phlegivirids. This may suggest that scaffolds 31 and 27 represent two different megabirnavirids, since some unannotated megabirna-like short contigs were also present in the dataset. We did not find any sequence that is similar to the P3 and P4 proteins encoded by the dsRNA2 of the characterized megabirnavirids [52,53].

RsTLV1 possesses two coding regions situated at two different ORFs and is translated in a –1-frameshift due to a shifty heptamer found at nucleotides 5038–5044 (in scaffold 19 and 21), similarly to many other members of the order *Ghabrivirales* [46] (Figure 5A). While RsTLV1 ORF2 encodes the viral RdRP, ORF1 encodes a hypothetical protein with no similarity to known proteins in the NCBI database.

The phylogenetic analysis revealed that all the abovementioned viruses belong to the suborder *Alphatotivirinae* in the order *Ghabrivirales*. RsPhV1 to RsPhV7 (probably also RsPhV8, scaffold 40) belong to the same family, *Phlegiviridae* (Figure 5B). According to the species demarcation for the order *Ghabrivirales*, viruses with ≥70% RdRP sequence identity belong to the same species. Each of the three pairs—RsPhV1 and RsPhV2 (94% RdRP sequence identity), RsPhV5 and RsPhV6 (90% RdRP sequence identity), and RsPhV3 and RsPhV8 (91% RdRP sequence identity)—represents the same species. However, the three sets belong to three distinct species. The RdRP sequence of RsPhV7 shows a 30% identity to its closest relative, PgLV1, classifying RsPhV7 as a new species under the family *Phlegiviridae*. On the other hand, RsMBV2 and its relatives (Pterostylis megabirnavirus-like, a 1.9K bp partial cds) may belong to the family *Megabirnaviridae* (Table 1). The analysis of the two RsTLV1 variants (scaffolds 19 and 21) showed an 86% sequence identity, suggesting that they belong to the same species. Together with other mycoviruses, they form a distinct clade far from any other groups suggesting that they belong to a different viral family, which we propose as “*Rhitotiviridae*” (Figure 5B).

### 3.6. Viruses in the Order Durnavirales, Phylum Pisuviricota

A total of seven scaffolds (4, 11, 18, 95, 428, 100, and 112) appeared to represent five novel viruses in the order *Durnavirales* (Table 1), tentatively designated as Rhizoctonia solani hypovirus 4 (RsHV4, scaffold 4), Rhizoctonia solani hypovirus 5 (RsHV5, scaffold 11), Rhizoctonia solani hypovirus 6 (RsHV6, scaffold 18), Rhizoctonia solani partitivirus 20 (RsPV20 dsRNA1 and dsRNA2, scaffolds 95 and 428), and Rhizoctonia solani partitivirus 19 (RsPV19 dsRNA1 and dsRNA2, scaffolds 100 and 112) respectively. Scaffold 38, slightly shorter than scaffold 18, also appeared to represent RsHV6, while scaffold 34 seems to be a truncated contig of an RsHV6 variant with a 76% nucleotide sequence similarity and an 87% amino acid sequence identity to scaffold 18. Moreover, we sequenced new virus isolates that are closely related to those that have been published previously (Table 1). BLASTx showed that the scaffolds 92 and 102 are the genomic segments of a known partitivirus: Rhizoctonia solani partitivirus SM03 [54,55]. We also found that scaffold 66 is one of the segments of Rhizoctonia solani dsRNA virus 1, a curvulavirid [56].

**Figure 6:**
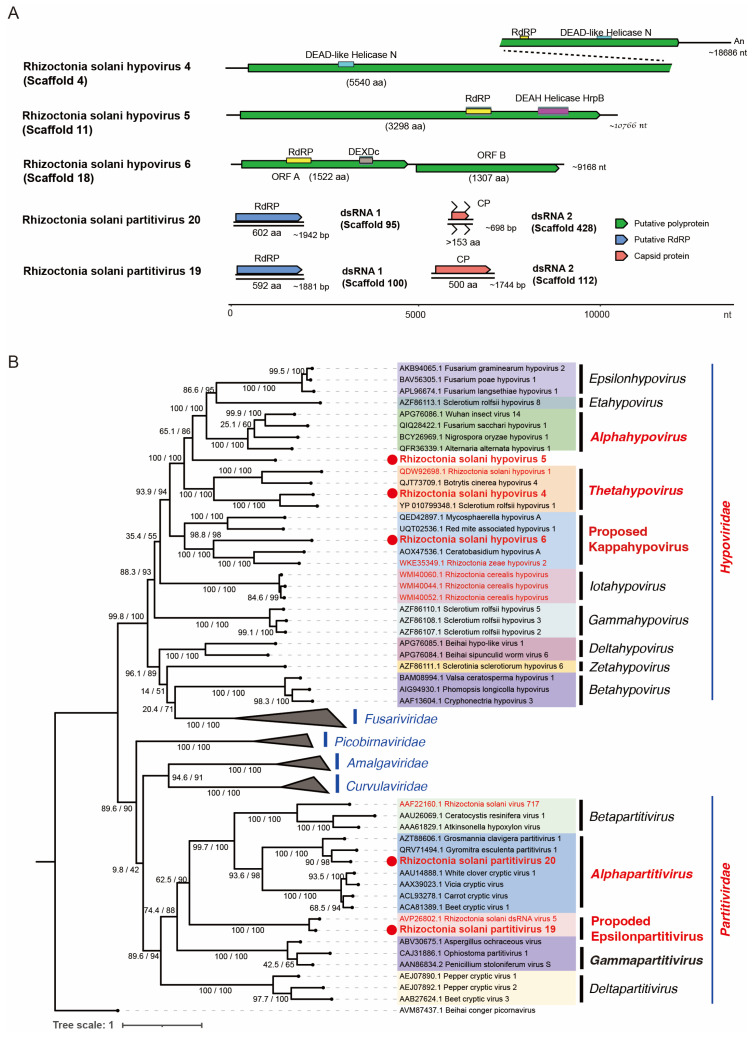
Genome organization and phylogenetic relationship of the newly detected viruses belonging to the order *Durnavirales.* (**A**) The schematic diagram of the genomes of the viruses discovered in this study. Three hypovirids and two partitivirids are shown. (**B**) The maximum likelihood tree generated based on the RdRP sequences. Different genera are shown by different colors. The ultrafast mode was used to construct the tree with 10,000 replicates calculating the bootstrap percentage and SH-aLRT, with rtREV+F+R10 as the substitution model. Beihai conger picornavirus (an unclassified conger eel picorna-like virus, order *Picornavirales*) was used as the outgroup.

The genome architecture of RsHV4 shows one large ORF (5540 amino acids) encoding a viral polyprotein with at least three identifiable domains for RdRP and two DEAD-like helicase domains (Figure 6A). A simple BLASTp of the protein domains showed that the helicase proximal to the C-terminal end of the polyprotein has a high sequence identity (79%) to that of Sclerotium rolfsii hypovirus 1 [57]. The dual helicase domains have been reported previously from other fungal viruses such as Rhizoctonia solani hypovirus 2 [19], although its biologic significance remains to be explored. The genome of RsHV5 includes a single large ORF polyprotein (3298 amino acids) with RdRP and the helicase domains of the DEAD-like helicase superfamily (Figure 6A), similarly to other hypoviruses [58]. RsHV6 has a peculiar two-ORF genome organization (Figure 6A), similar to that of Rhizoctonia zeae hypovirus 2 (RzHV2) [23]. The 5′-proximal ORF encodes a protein of 1522 amino acids with the RdRP and the helicase domains, while the 3′-proximal ORF (second ORF) encodes another protein with 1307 amino acids that has no sequence similarity to other viral proteins, except for the second ORF product of RzHV2 with a weak sequence similarity (Figure 6B). The classical hypoviruses with a two-ORF genome utilize a termination/re-initiation or stop/restart translation mechanism to express their downstream ORFs from genome-sized messenger RNA (mRNA). Typically, in hypoviruses, two ORFs are linked by a tentative stop/restart pentameric sequence, UAAUG, which serves as the stop codon (UAA) for the first ORF and the start codon (AUG) for the second ORF [59]. However, the ORFB of RsHV6 is situated 232 nucleotides downstream of this pentanucleotide. It is unknown how RsHV6 ORFB is expressed (Figure 6A).

Apart from the hypovirids, we also found two partitivirus strains (RsPV20 and RsPV19) with two dsRNA segments each encoding for RdRP and CP (Figure 6A, Table 1 and Table S2). The RsPV20 dsRNA1 of 1942 bp appears to be coding-complete, encoding RdRP, while dsRNA2 (698 bp) is a partial genomic sequence thought to encode CP. RsPV19 is likely to have a coding-complete genome architecture with dsRNA1 and dsRNA2 encoding for RdRP (592 amino acids) and CP (500 amino acids), respectively (Figure 6A).

We analyzed the taxonomic placement of each virus in this study by constructing a phylogenetic tree to determine specific clade associations (Figure 6B). The genus demarcation criteria currently set for the family *Hypoviridae* depends on taxonomic clustering in a phylogenetic tree based on the sequence of the entire length of its genome. According to this, RsHV4 belongs to *Thetahypovirus*, while RsHV6 clusters with a unique set of viruses that we propose to designate as “*Kappahypovirus*” to accommodate this separate clade [58]. Since RsHV5 does not cluster with the known members of the established genus in the family (Figure 6B), further analysis will be required. Meanwhile, sequence similarity to the closest relative ranges from 28% up to 49% for RsHV4, RsHV5, and RsHV6. There are currently nine genera within *Hypoviridae*, and the addition of “*Kappahypovirus*” is supported by a high bootstrap score (97.4%) and a well-supported SH-aLRT score of 99% (Figure 6B). RsPV20 and RsPV19 appear to represent two new species in the genera *Alphapartitivirus* and the proposed genus “*Epsilonpartitivirus*”, respectively, based on the species demarcation criteria for *Partitiviridae* [60,61].

### 3.7. Viruses in the Families Botourmiaviridae and Mitoviridae within Orders Ourlivirales and Crypavirales, Phylum Lenarviricota

**Figure 7:**
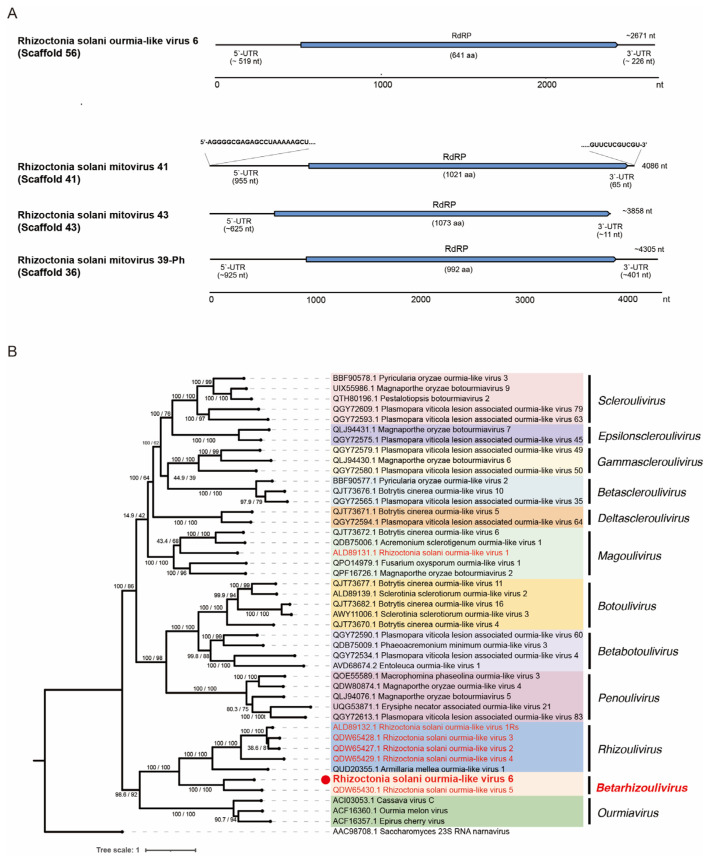

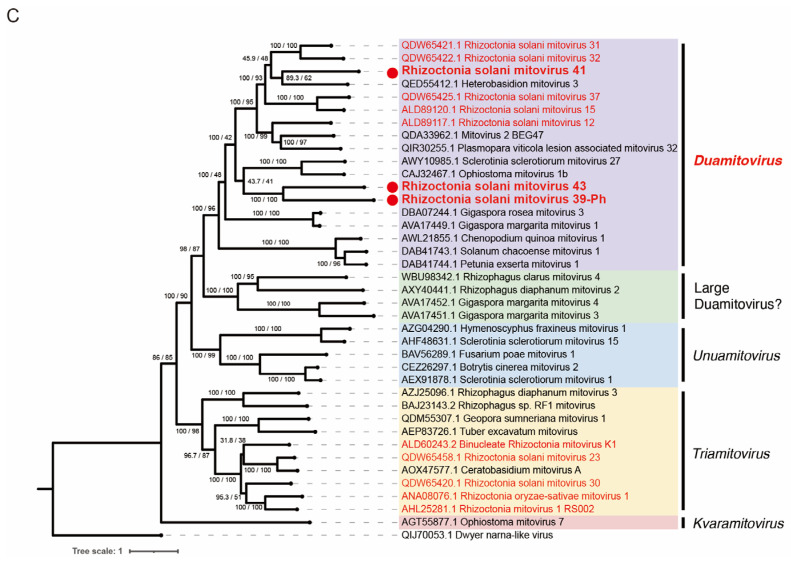
Illustration of the genomes and phylogenetic relationship of newly detected viruses belonging to the orders *Ourlivirales* and *Crypavirales*. (**A**) The schematics of the genomes of one ourmiavirid and three mitovirids. While the genome of *Rhizoctonia solani* mitovirus 41 was completely sequenced, those of the other three members of the phylum *Lenarviricota* appeared to be coding-complete. The maximum likelihood trees based on the RdRP sequences for (**B**) ormiavirids and (**C**) mitovirids. The trees were constructed using the IQ-TREE online server with the ultrafast mode and 10,000 replicates to calculate the bootstrap percentage, and the rtREV+F+R5 (**B**) or mtInv+F+R6 substitution model (**C**) based on the Bayesian information criterion predicted by ModelFinder. The outgroup used is the *Saccharomyces* 23S RNA narnavirus (a proto-type of narnavirids, order *Wolframvirales*) (**B**) or *Dwyer narna*-like virus (an unclassified narnavirid of rabbits or associated ectoparasites, order *Wolframvirales*) (**C**).

A single contig sequence (scaffold 56) is similar to the (+)ssRNA viruses within the family *Botourmiaviridae* (order *Ourlivirales*), and thus we tentatively designated this candidate virus as Rhizoctonia solani ourmia-like virus 6 (RsOLV6; Table 1). RsOLV6 is approximately 2.7 kb and encodes a single ORF of RdRP (641 amino acids; Figure 7A). The phylogenetic analysis showed that RsOLV6 clusters with Rhizoctonia solani ourmia-like virus 5 (RsOLV5), a sole member of the genus *Betarhizoulivirus*, with strong node support (Figure 7B). The sequence comparison between RsOLV6 and its closest relative (RsOLV5) shows a 46% amino acid sequence identity, which is below the species demarcation criteria in the genus (>90% amino acid sequence identity) [62]. These two *R. solani* viruses, RsOLV6 and RsOLV5, have long untranslated regions (UTRs; 519 and 919 nucleotides, respectively), although the genome of RsOLV6 is much shorter than that of RsOLV5 (5234 nucleotides) [18].

The scaffolds 36, 41, and 43 likely represent three mitovirids: Rhizoctonia solani mitovirus 41 (RsMV41), Rhizoctonia solani mitovirus 43 (RsMV43), and Rhizoctonia solani mitovirus 39-Ph (RsMV39-Ph), respectively (Table 1). We have provided the complete sequence of RsMV41, including the termini determined through RLM-RACE. Their genomes possess a single ORF that encodes the RdRP of 992 amino acids (RsMV39-Ph), 1021 amino acids (RsMV41), and 1073 amino acids (RsMV43), with long 5′-UTR and short 3′-UTR regions (Figure 7A). The amino acid tryptophan (Trp) is often encoded by the UGA codon in the mitochondrial genetic code; it serves as a stop codon in the cytosolic or standard genetic code, making UAG and UAA the only stop codons in the mitochondria of fungi [63]. The visual inspection of the translated ORF sequences of RsMV39-Ph, RsMV41, and RsMV43 shows that there is no in-frame UGA codon that codes for Trp, suggesting that these mitoviruses may also replicate in the cytosol, as described previously [64]. However, this may reflect that UGA(Trp) codons are rare and not favored in *R. solani* mitochondrial genes [65].

Phylogenetic analysis showed that three mitoviruses (RsMV39-Ph, RsMV41, and RsMV43) clustered with the members of the genus *Duamitovirus* with a strong node support, and adjacent to another clade with members with a large ORF in arbuscular mycorrhizal fungi “Large Duamitovirus” (Figure 7C) [66]. RsMV39-Ph, RsMV41, and RsMV43 show varying degrees of amino acid sequence similarity to other mitovirids, and very high sequence identity (94~97%) to recently deposited mitovirus-related sequences (Rhizoctonia solani mitovirus 125 and 129, and Hangzhou mito-like virus 3 from *R. solani* strains or plant metagenome analysis in China). These represent new species of the genus *Duamitovirus*.

## 4. Discussion

### 4.1. First R. solani Virome Report from a Philippine Fungal Population

Rice sheath blight was first discovered in Japan in 1910 and has since been reported in various parts of the world [67]. Sheath blight caused by *R. solani* (a basidiomycete) induces major economic losses due to a yield reduction of up to 48% in different rice-producing countries. The virome analyses of *Rhizoctonia* spp., including *R. solani* have been performed mainly to better understand the virus diversity and to search for potential virocontrol agents. In terms of mycovirus diversity, a single *R. solani* strain can host up to several—as many as 27—viruses [18]. So far, the identified mycoviruses reported from *R. solani* are classified in or associated with at least 10 (+)ssRNA virus families and 2 dsRNA virus families, as well as 2 (–)ssRNA mycovirus groups related to the orders *Serpentovirales* and *Bunyavirales* [18,21]. Approximately 100 viruses have been reported for this or related fungal species, and the genomic information of some representative viruses has been summarized by Abdoulaye et al. [21]. To date, an increasing number of novel as-yet-unclassified mycoviruses in *Rhizoctonia* spp. have been described using NGS techniques [18,21,68,69].

In this study, we reported, for the first time, a fungal virome analysis of a Philippine *Rhizoctonia solani* population. A dsRNA-based survey for the potential virus infection of a total of 40 Philippine *R. solani* strains revealed an extremely high (100%) prevalence rate based on a conventional dsRNA assay (Figure S3). The NGS analysis of the three representative *R. solani* strains (Tar-Rs-3, Ilo-Rs-6, and Tar-Rs-5) allowed us to identify a number of virus-related sequences (at least 35) apparently belonging to 22 new species and 3 previously established species (Table 1). These newly discovered viruses or virus-like sequences from Philippine *R. solani* strains likely represent coding-complete or nearly complete genomic sequences. For two viruses (RsPhV1 and RsMV41), we employed the RLM-RACE analysis to determine the complete genomic sequences. The viruses characterized in this study are related to several (+)ssRNA virus families such as *Alphatetraviridae*, *Benyviridae*, *Hypoviridae*, *Endornaviridae*, *Botourumiaviridae*, and *Mitoviridae*, as well as the dsRNA virus families *Megabirnaviridae*, *Partitividae*, and the proposed family “*Phlegiviridae*” [46,50]. However, we did not detect any (–)ssRNA viruses. We considered some of the (+)ssRNA viruses to be members of the proposed families “*Mycobenyviridae*” and “*Mycoalphaviridae*.” In addition, for two unassigned groups, we newly proposed the creation of the new family “*Rhitotiviridae*” (after Rhizoctonia toti-like viruses) in the suborder “*Alphatotivirinae,*” and the new genus “*Kappahyopovirus*” in the family *Hypovoviridae* (according to the current genus nomenclature system of the family). Thus, this study has expanded the virome of *Rhizoctonia* spp., specifically *R. solani* populations.

Among the three *R. solani* strains tested, the two *R. solani* strains Tar-Rs-3 and Ilo-Rs-6 from different islands, Luzon (Tarlac) and Visayas, respectively, shared seven virus infections (Figure 2B). In contrast, the third strain Tar-Rs-5 from Luzon shares only two viruses with these strains, even with the other strain from the same island (Tar-Rs-3); 10 other viruses of Tar-Rs-3 were undetectable in the other two tested strains. These findings suggest that there is some overlap of the mycoviral population in host fungi between different regions (islands), while sufficient diversity can also be observed in the populations within the same region (island). The large-scale sampling of fungal strains from the rice-producing areas of the major islands in the Philippines is necessary, and the identification of the mycoviruses infecting them, as well as the remaining strains used for dsRNA screening, should lead to a better understanding of the Philippine *R. solani* virome.

### 4.2. High Prevalence Rate of Virus Infection for Rhizoctonia Strains

When assayed for potential virus infections by the gel electrophoresis of dsRNA isolated via conventional cellulose column chromatography, all 40 fungal strains we tested appeared to be infected with mycoviruses (Figure S3). The virus prevalence rate generally varies depending on factors such as the host fungal species, fungal sampling procedures, virus detection methods, and fungal maintenance conditions. Although we tested a relatively small number of fungal samples (a total of 40 strains), the estimated 100% prevalence rate is extremely high compared with the previously reported rates for *Rhizoctonia* spp. For example, Zheng et al. [70] reported only 16 of 43 (37%) *R. solani* strains from nine provinces in China were infected with mycoviruses, while almost half of the *R. solani* strains from the USA (5/12) carried dsRNA [71]. However, in agreement with our findings, Zanzinger et al. [72] also reported an almost 100% infection rate (49/50) of the field isolates of *R. solani*. Bharathan et al. [73] also reported that all 36 *R. solani* field isolates belonging to nine AGs and originating from different countries (USA, Australia, Japan, Mexico, and South Africa) were infected with mycoviruses. High prevalence rates have also been observed in *R. solani* isolated from different crops such as beets [26] and potatoes [23]. NGS approaches also revealed high prevalence rates of *R. solani* isolated from turfgrass such as Japanese lawngrass (8/8, 100%) [18].

### 4.3. Multiple Infections of Single R. solani Strains and Their Stability

Almost all of the fungal strains we tested in this study were infected by multiple RNA viruses, as previously reported for *R. solani* and other *Rhizoctonia* spp. [18,23,69]. All the tested fungal strains showed at least 15 distinguishable dsRNA banding patterns, suggesting multiple virus infections (Figure 1B and Figure S3). In addition, we detected hyper-coinfection by mycoviruses (up to 20 viruses) based on NGS analysis in at least three tested fungal strains (Figure 2B). Our findings for *R. solani* infection are not surprising, as other researchers have reported infections with a large number of viruses: 27 viruses harbored in an *R. solani* isolate [18] and 39 viruses in a *Rhizoctonia cerealis* isolate [74]. It remains unknown what factors are responsible for the high prevalence rates and multiple infections in *Rhizoctonia* spp. Overwintering sclerotia in or on the soil is the primary source of rice sheath blight infection, consistent with the nature of *R. solani* (a basidiomycete). Moreover, sexual basidiospores are important for the infection cycle, dispersal, and genetic diversification of this fungus [75,76]. Several factors may account for this very high virus prevalence rate and probably frequent multiple infections. First, the viruses in *Rhizoctonia* are likely efficiently transmitted through sclerotia and basidiospores. The investigation of how efficiently *Rhizoctonia* viruses are transmitted vertically, particularly through sclerotia, is limited to a few studies [21]. Second, those viruses are likely transmitted horizontally between AG groups or species in nature more efficiently than expected. There is circumstantial evidence for horizontal transmission between the different AG groups of *R. solani*; different strains of a single virus species with greater than 95% sequence identity have been detected from multiple AGs [18,25] and related *Rhizoctonia* species [23]. This study also strongly suggested the inter-AG transmission of a partitivirus (RsPV-SM03) between AGI-IA and AG4 HGIII (Table 1) [77]. It may be possible that viruses move between different AG groups via direct hyphal contact or indirectly through a biological vector-mediated route involving uncharacterized invertebrate vectors or plants, as proposed previously [78,79]. Inter-AG-group and interspecies transmission may occur more frequently under natural conditions than under laboratory in vitro conditions, as observed for virus/ascomycete combinations [80]. Third, the viruses that infect *Rhizoctonia* spp. are likely to be maintained stably and very difficult to eliminate even with the use of antiviral drugs. Our attempts to eliminate viruses from the *R. solani* strains Tar-Rs-3, Ilo-Rs-6, and Tar-Rs-5 using different methods with antiviral drugs failed. Failures in similar attempts have been reported previously for different virus/*Rhizoctonia* spp. combinations [24]. Of note, some viruses have been successfully eliminated by different methods [19,41,81]. These lost viruses may have much lower viral fitness than the retained viruses.

There are different types of virus/virus interactions in single fungal host individuals [82,83], as exemplified by synergistic and antagonistic interactions. However, no such interactions have been demonstrated for *Rhizoctonia* spp. The potential for virus/virus interactions in the fungal strains we tested, particularly the Tar-Rs-3, Ilo-Rs-6, and Tar-Rs-5 strains harboring >10 viruses, is worth exploring in the future. In fact, the TAR-Rs-3 strain harbored at least 20 unique viruses belonging to 11 families (Figure 2A). The exploration of virus/virus interactions virus in *R. solani* requires the development of versatile virus elimination and introduction protocols for each co-infecting virus.

## Figures and Tables

**Table 1:** A list of virus-assembled sequences from the *Rhizoctonia solani* virome analysis.

Virus Abbreviation ^a^	Segments	Scaffold No. ^b^	Length	Read	Accession No.	BLASTx Top Hit Virus ^i^	Identity (%)
			(nt or bp)	Counts			
RsALV4		25	7992	1200	MW596323	Rhizoctonia cerealis alphavirus-like virus	47.06
RsALV5		20	9046	5722	PP262119	Rhizoctonia cerealis alphavirus-like virus	46.45
RsBLV2		22	8463	31,965	MW574435	Bemisia tabaci beny-like virus 6	34.64
RsBLV3		26	7808	707	MW574436	Rhizoctonia solani beny-like virus 1	46.53
RsBLV4		269	1026	73	MW596324	Rhizoctonia solani beny-like virus 1	69.66
RsEV8		5	18,645	2183	MW574437	Rhizoctonia solani endornavirus 1	44.57
RsEV9		6	14,601	3299	MW574438	Rhizoctonia solani endornavirus 7	38.6
RsEV1 ^c^		2	19,876	2652	MW596327	Rhizoctonia solani endornavirus 1	90.79
		3	19,860	3552	MW596329	Rhizoctonia solani endornavirus 1	82
		1	20,088	5629	MW596328	Rhizoctonia solani endornavirus 1	82.14
RsPhV3 ^d^		15	10,433	2637	MW574447	Rhizoctonia solani dsRNA virus 7	45.46
RsPhV1 ^e^		9	12,389	1913	MW596343	Rhizoctonia solani dsRNA virus 7	60.43
RsPhV2 ^e^		10	10,888	2040	MW596348	Rhizoctonia solani dsRNA virus 7	60.09
RsPhV5 ^f^		7	11,460	2911	MW596349	Rhizoctonia solani dsRNA virus 7	58.88
RsPhV6 ^f^		8	11,164	2386	MW596350	Rhizoctonia solani dsRNA virus 7	59.1
RsPhV7 ^g^		24	7994	787	MW574448	Phlebiopsis gigantea large virus 1	30.49
RsPhV8 ^g^		40	4180	16,318	MW574449	Lentinula edodes mycovirus HKB	37.28
RsPhV4 ^d^		14	10,449	1987	MW596344	Rhizoctonia zeae RNA virus 1	42.52
RsMBV2	dsRNA1	31	4866	676	MW560186	Pterostylis megabirnavirus-like RdRP	70.26
RsMBV3 ^g^	dsRNA1?	27	6649	3867	MW596351	Rhizoctonia zeae megabirnavirus 1 CP	26.28
RsTLV1		19	9046	32,346	MW574450	XiangYun toti-like virus 7	30.82
		21	9040	22,486	MW574451	XiangYun toti-like virus 7	31.03
RsRV ^c^		16	10,190	11,311	MW596346	Rhizoctonia solani RNA virus	85.19
		13	10,453	8801	MW596347	Rhizoctonia solani RNA virus	85.41
		12	10,467	7239	PP334487	Rhizoctonia solani RNA virus	85.98
RsYnV1^g^		377	785	46	PP334490	Rhizoctonia zeae yadonushi virus 2	53.01
RsHV4		4	18,697	86,912	MW574439	Sclerotium rolfsii hypovirus 1	59.07
RsHV5		11	10,766	1538	MW574440	Nigrospora oryzae hypovirus 1	33.16
RsHV6		18	9168	10,872	MW574441	Rhizoctonia zeae hypovirus 2	33.16
		38	4235	904	MW574442	Rhizoctonia zeae hypovirus 2	27.61
		34	4489	4949	MW574443	Rhizoctonia zeae hypovirus 2	27
RsPV20	dsRNA1	95	1942	2586	MW596340	Grosmannia clavigera partitivirus 1 RdRP	66.83
	dsRNA2	428	698	50	PP334488	Grosmannia clavigera partitivirus 1 CP	41.49
RsPV19	dsRNA1	100	1881	286	MW596341	Rhizoctonia solani dsRNA virus 5	82.6
	dsRNA2	112	1744	242	PP262120	Rhizoctonia solani dsRNA virus 5	70.53
RsPV-SM03 ^c^	dsRNA1	92	1976	8112	MW596342	Rhizoctonia solani partitivirus SM03	98.96
	dsRNA2	102	1852	1624	PP334489	Rhizoctonia solani partitivirus SM03	96.72
RsRV1 ^c^		66	2321	534	MW596338	Rhizoctonia solani dsRNA virus 1	98.39
RsMV39-Ph ^h^		36	4305	1,374,127	MW574444	Rhizoctonia solani mitovirus 15	59.17
RsMV41		41	4086	2,530,917	MW596335	Binucleate Rhizoctonia mitovirus 8	62.14
RsMV43		43	3858	4,698,115	MW596337	Binucleate Rhizoctonia mitovirus 16	72.66
RsOLV6		56	2671	306	MW574434	Rhizoctonia solani ormia-like virus 5	42.84

^a^ see Table S2 for the full names of viruses and other information. ^b^ scaffolds were obtained by assembly analysis using the SPAdes assembly program. ^c^ viruses previously published or with similar entries in GenBank. ^d–f^ those with the same letters are likely to belong to the same species. ^g^ the RdRP CDS sequences of the novel viruses are unconfirmed or largely incomplete. ^h^ the entry with the same virus name (RsMV39), but probably not the same virus species, is presented in the database. ^i^ note that no rice-infecting viruses were returned as top hits.

## Data Availability

The sequences reported in the present manuscript have been deposited in the GenBank database under accession numbers listed in Table 1. All the other data are available in the manuscript and Supplementary dataset.

## Supplementary Materials

The following supporting information can be downloaded at https://www.mdpi.com/article/10.3390/v16071152/s1, Table S1: Characterization of *Rhizoctonia solani* isolates collected from various rice fields in Luzon, Visayas, and Mindanao, Philippines. Table S2: Viral sequences obtained in this study and their details. Table S3: List of primer sets for RT-PCR analysis. Table S4: List of primers for RLM-RACE. Figure S1: Diagram of fungal isolation. Figure S2: Colony morphology of the *R. solani* collection grown on PDA and oatmeal plates. Figure S3: The dsRNA banding profile of the 40 *R. solani* strains.
