# Supplementary material for: Metatranscriptomic Sequencing of Sheath Blight-Associated Isolates of Rhizoctonia solani Revealed Multi-Infection by Diverse Groups of RNA Viruses
Source: Viruses. 2024 Jul 17;16(7):1152. doi: 10.3390/v16071152 (PMC11281561; doi:10.3390/v16071152)
Supplement: Supplementary file 1 [file viruses-16-01152-s001.zip › viruses-3082087-supplementary.pdf]

Supplementary Information for

Metatranscriptomic Sequencing of Sheath Blight-Associated Isolates of *Rhizoctonia solani* Revealed Multi-Infection by Diverse Groups of RNA Viruses

Michael Louie R. Urzo[1, 2], Timothy D. Guinto[1, 2], Ana Eusebio-Cope[3, *], Bernard O. Budot[4], Mary Jeanie T. Yanoria[2], Gilda B. Jonson[2], Masao Arakawa[5], Hideki Kondo[6, *], and Nobuhiro Suzuki[6, *]

[1]Microbiology Division, Institute of Biological Sciences, College of Arts and Sciences, University of the Philippines Los Baños, Laguna 4031, Philippines

[2]Traits for Challenged Environments Unit, Rice Breeding Innovations Department, International Rice Research Institute (IRRI), UP Los Baños, College, Los Baños Laguna 4031, Philippines

[3]Fit-for-Future Genetic Resources Unit, Rice Breeding Innovations Department, International Rice Research Institute (IRRI), UP Los Baños, College, Los Baños Laguna 4031, Philippines

[4]Institute of Weed Science, Entomology, and Plant Pathology, College of Agriculture and Food Science, University of the Philippines Los Baños, Laguna 4031, Philippines

[5]Faculty of Agriculture, Meijo University, Nagoya, 468-8502, Japan

[6]Plant-Microbe Interactions Group, Institute of Plant Science and Resources (IPSR), Okayama University, Chuo 2-20-1, Kurashiki, 710-0046 Okayama, Japan

***Corresponding author**

Lead contact: Dr. Nobuhiro Suzuki

ORCID ID, http://orcid.org/0000-0003-0097-9856

Agrivirology Laboratory

Institute of Plant Science and Resources

Okayama University

Kurashiki, Okayama 710-0046, Japan

Tel. 81(86) 434-1230

Fax. 81(86) 434-1232

e-mail. nsuzuki@okayama-u.ac.jp

Data information:

Supplemental figures, 3.

Supplemental tables, 4.

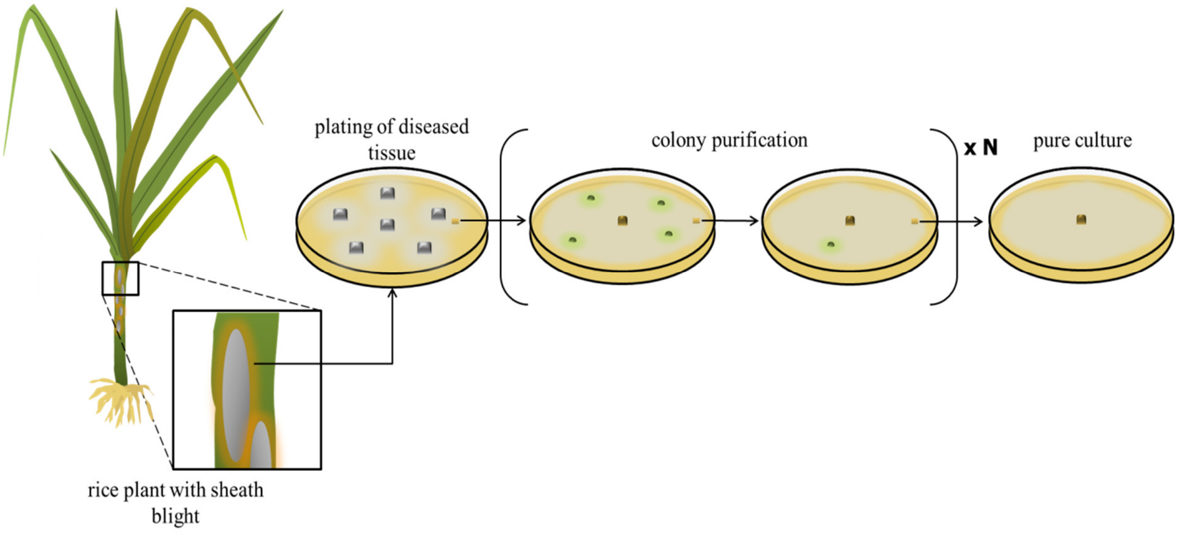

Figure S1. Diagram of fungal isolation. Isolation of the pathogen involved inoculation of plant infected tissue in culture and serial transfer for purification.

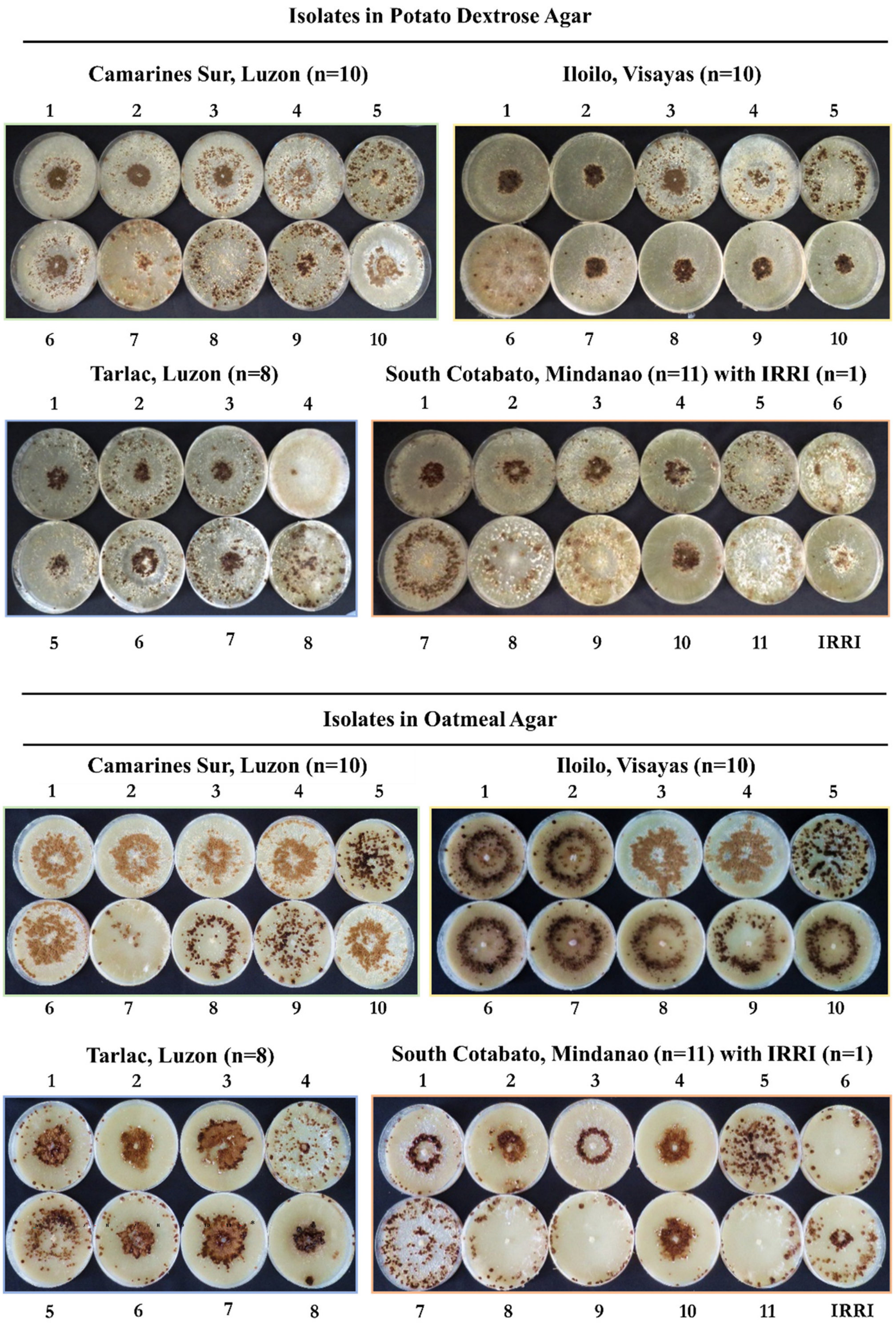

Figure S2. Colony morphology of the *R. solani* collection grown on PDA and oatmeal plates. The fungal strains were cultured in 90-mm petri plates for 10 days in constant darkness at 28 °C before being photographed; then, colony morphology was compared. See Table S1 for the details of the fungal strains.

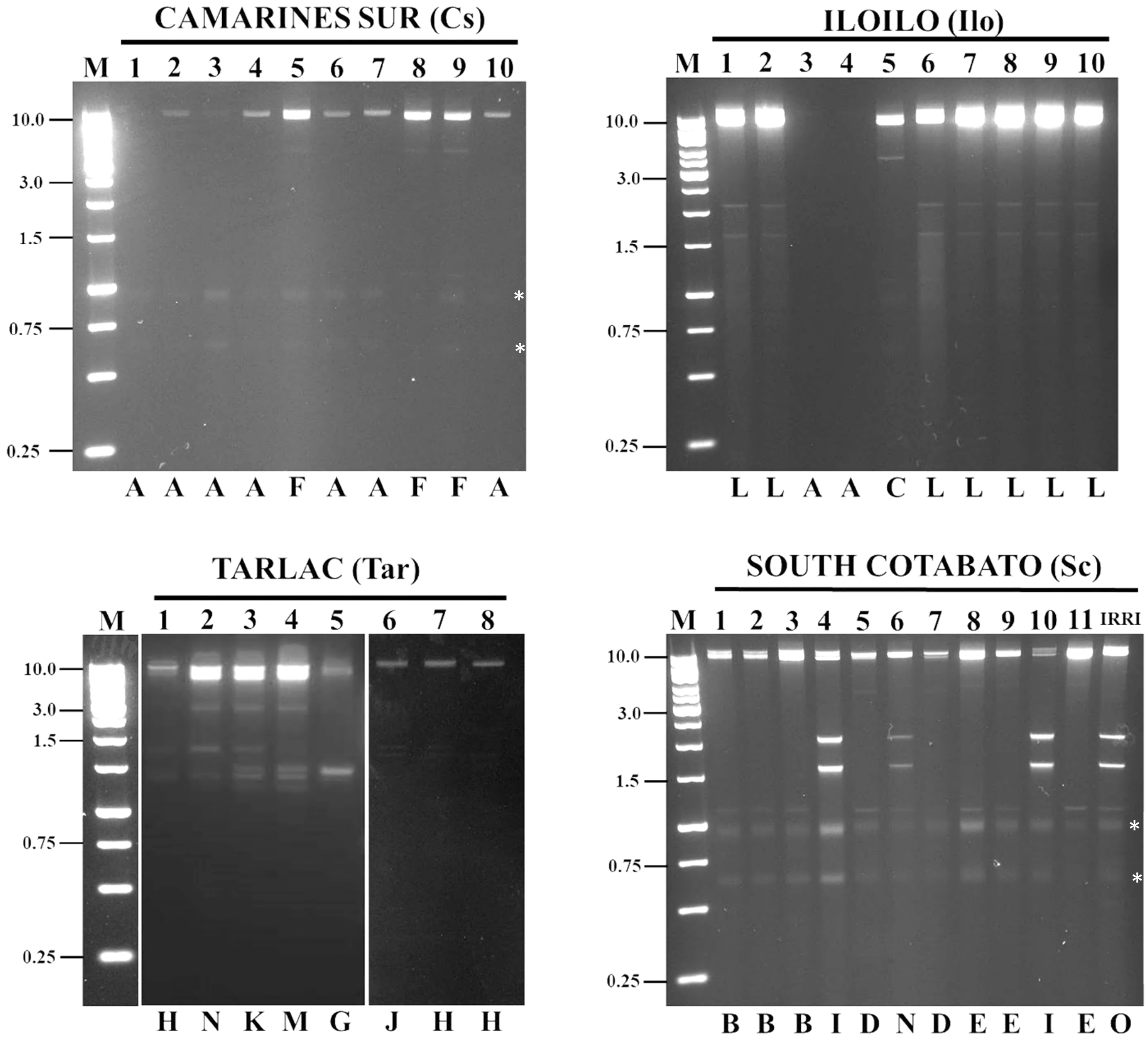

Figure S3. The dsRNA banding profile of the 40 *R. solani* strains. The purified dsRNA fractions of the fungal strains were subjected to 1.4% agarose gel electrophoresis followed by ethidium bromide staining. The asterisk indicates rRNA bands. The dsRNA banding profile was categorized based on the results of the dsRNA patterns shown in here and additional dsRNA analyses, and summarized in Figure 1B.
